# No Significant Effects of IL-23 on Initiating and Perpetuating the Axial Spondyloarthritis: The Reasons for the Failure of IL-23 Inhibitors

**DOI:** 10.3389/fimmu.2022.818413

**Published:** 2022-02-10

**Authors:** Hua Zhang, Han-Lei Jiang, Sheng-Ming Dai

**Affiliations:** Department of Rheumatology & Immunology, Shanghai Jiao Tong University Affiliated Sixth People’s Hospital, Shanghai, China

**Keywords:** axial spondyloarthritis, IL-23, Th17 cells, IL-17, IL-23 inhibitors, ankylosing spondylitis

## Abstract

Axial spondyloarthritis (axSpA) is comprised of ankylosing spondylitis (AS) and non-radiographic axSpA. In recent years, the involvement of the interleukin (IL)-23/IL-17 axis in the pathophysiology of axSpA has been widely proposed. Since IL-23 is an upstream activating cytokine of IL-17, theoretically targeting IL-23 should be effective in axSpA, especially after the success of the treatment with IL-17 blockers in the disorder. Unfortunately, IL-23 blockade did not show meaningful efficacy in clinical trials of AS. In this review, we analyzed the possible causes of the failure of IL-23 blockers in AS: 1) the available data from an animal model is not able to support that IL-23 is involved in a preclinical rather than clinical phase of axSpA; 2) Th17 cells are not principal inflammatory cells in the pathogenesis of axSpA; 3) IL-17 may be produced independently of IL-23 in several immune cell types other than Th17 cells in axSpA; 4) no solid evidence supports IL-23 as a pathogenic factor to induce enthesitis and bone formation. Taken together, IL-23 is not a principal proinflammatory cytokine in the pathogenesis of axSpA.

## Introduction

Axial spondyloarthritis (axSpA) comprised of ankylosing spondylitis (AS) and non-radiographic axSpA (nr-axSpA) is an inflammatory disease of the axial skeleton. Historically, therapies of AS have been limited to mainly physiotherapy and non-steroidal anti-inflammatory drugs (NSAIDs), while conventional immunosuppressants or disease-modifying anti-rheumatic drugs may only be helpful in concurrent peripheral joint involvement. In the last two decades, the major therapeutic breakthrough has been the advent of biologic drugs TNF inhibitors (TNFi), which have shown good efficacy in patients who were non-responders or insufficient responders to NSAIDs, with reduction of pain, stiffness, and physical handicap. However, up to 40% of axSpA patients do not demonstrate a meaningful clinical response to TNFi, and some patients lose response during treatment (secondary therapeutic failure) (1). Further, TNFi may be contraindicated for certain patients. So there is still a need for an additional class of biologic.

The discovery of the interleukin (IL)-23/IL-17 pathway revealed key molecules involved in the pathophysiology of axSpA. When IL-23 binds to its receptor IL-23R, the complex recruits JAK2 and Tyk, members of the Janus family of tyrosine kinases, which in turn mediate the activation of the IL-23/IL23R and, eventually, the phosphorylation of the downstream STAT3 (2). IL-23 stimulates the differentiation and expansion of Th17 cells *via* IL-23R and induces the release of molecules such as IL-17 or IL-22, eventually activating the “effector cells” keratinocytes (3, 4), B cells, osteoclast precursors, and macrophages (5, 6). Mounting evidence demonstrates that the IL-23/IL-17 axis appears to be one of the main pathways involved in the development of axSpA. It is reasonable that the inhibition of IL-23 and IL-17 should be an alternative choice for the treatment of axSpA.

Clinical trials performed with the IL-17-blocking monoclonal antibodies secukinumab, ixekizumab, bimekizumab, and netakimab have clearly shown significant reductions in the signs and symptoms of patients with radiographic axSpA or AS (7–10) and of patients with non-radiographic axSpA (11, 12). Furthermore, long-term treatment with secukinumab retarded structural progression in AS (13).

But drugs blocking IL-23 lacked efficacy in the treatment of axSpA. For instance, ustekinumab, an IL-12/IL-23 inhibitor, was assessed in axSpA in three trials, but the latter two were discontinued because of failure in the first trial (14). Risankizumab, an IL-23p19 inhibitor, failed in AS to meet the primary endpoint (15). The converse efficacy of IL-17A inhibition and IL-23 inhibition in AS suggests that IL-17A rather than IL-23 is the major cytokine mediating disease pathogenesis in axSpA. Targeting the two members of the IL-23/IL-17 axis, both anti-IL-17 and anti-IL-23 treatment have shown beneficial effects in psoriasis and PsA (16–18), while IL-17 inhibitors rather than IL-23 inhibitors are effective in axSpA. Here, we discussed the possible causes for the therapeutic failure of IL-23 inhibition in axSpA.

## Is IL-23 Involved in Initiation Rather Than Perpetuation of Axial Spondyloarthritis?

IL-23 is primarily secreted by antigen-presenting cells such as macrophages and dendritic cells and along with other cytokines including IL-1 and IL-6 in tissues like the skin, intestinal mucosa, lungs, synovium, and brain. IL-23 expression is induced by stimulation of myeloid-derived cells with pathogen ligands, as well as other mediators such as prostaglandin E2 and proinflammatory cytokines. IL-23 is a pleiotropic cytokine critical for the differentiation, survival, and expansion of conventional (αβ) T cells and unconventional (γδ) T cells (19), which regulate a plethora of immune responses, and it can promote the polarization to IL-17-expressing cells.

Some researchers believed that one of the possible reasons why IL-23 inhibitors are ineffective in axSpA may be that IL-23 is pivotal for the initiation of disease but not involved in ongoing or persistent inflammation (6, 20–22). The HLA-B27/Huβ2m transgenic rats were immunized with heat-inactivated *Mycobacterium tuberculosis* to induce an experimental SpA model. By using the SpA model, van Tok et al. (20) reported that treatment with anti-IL-23R before the signs of arthritis/spondylitis onset could completely inhibit the development of spondylitis and peripheral arthritis, whereas treatment with anti-IL-23R 1 week after 50% arthritis incidence failed to reduce the incidence and the severity of experimental SpA. In contrast, anti-IL-17 treatment suppressed arthritis/spondylitis in both the initiating phase and established phase of the same SpA model and inhibited the periosteal new bone formation (23, 24). So these data seem to support that axSpA might be IL-23-dependent in the initiation of the disease but IL-23-independent once the disease is established, which is different from IL-17.

Collagen-induced arthritis (CIA) is a classical model of experimental arthritis mimicking rheumatoid arthritis (RA), which is characterized by synovitis, cartilage destruction, and bone erosion. In CIA mice, *IL-23p19* knockout protected the mice to develop clinical signs of disease and completely inhibited the development of joint and bone pathology (25). Neutralizing anti-IL-23p19 antibody (anti-IL23p19) treatment before the signs of arthritis onset significantly decreased arthritis score and histological severity in CIA mice, but the treatment lost the inhibitory effects when given after the onset of arthritis (26). These data also indirectly support that IL-23 only plays a pathological role in the initiating phase of inflammatory arthritis. However, IL-23p19 knockout did not prevent the onset of joint inflammation in a murine model of antigen-induced arthritis (27). Polyclonal anti-IL-23 antibody treatment after the onset of arthritis significantly decreased paw volume, synovial tissue inflammation, and bone destruction in CIA rats in a dose-dependent manner (28). These data do not support the above hypothesis that IL-23 may only play a pathogenic role in initiating arthritis.

Although animal models are widely used to test potential new therapies, there is no ideal animal model with high predictive values of therapeutic efficacy in human axSpA. It is easier for a compound/agent to exert beneficial efficacy in experimental arthritis than human arthritis. Moreover, it is easier to show the efficacy of protective treatment than therapeutic treatment in animal models of arthritis. For example, there are a huge amount of compounds to be demonstrated to have prophylactic efficacy to inhibit the development of arthritis in CIA models, including dietary ingredients/supplements (e.g., resveratrol, docosahexaenoic acid, and salmon proteoglycan) (29–31), countless herb extracts (e.g., *Acanthopanax senticosus* Harms extract, aqueous extract of *Trachyspermum ammi* seeds, and *Glycine tabacina* ethanol extract) (32–34), anti-inflammatory agents (e.g., ibuprofen, celecoxib, etodolac, indomethacin, and dexamethasone) (34–36). Vascular endothelial growth factor (VEGF) is a growth factor with important pro-angiogenic activity. Neutralizing anti-VEGF mAb was demonstrated to have prophylactic but not therapeutic actions in CIA mice (37). We cannot take for granted that these data support that VEGF is only involved in the initiation rather than persistence of RA. Since other studies demonstrated that expression levels of VEGF mRNA and protein were associated with severity of arthritis in CIA mice (38), clinical data supported that VEGF plays a pivotal role in the pannus formation/angiogenesis in rheumatoid synovium (39).

Evidence shows that a compound’s therapeutic efficacy rather than prophylactic efficacy in both CIA and adjuvant-induced arthritis (AIA) models has a more predictive value of clinical efficacy in patients with RA than efficacy from either model alone (40). IL-17 deficiency markedly suppressed joint inflammation and destruction in both CIA and streptococcal cell wall-induced arthritis models (41, 42). IL-17A gene transfer exacerbated synovial inflammation and bone loss before noticeable joint swelling was established in CIA mice (43). Both preventive and therapeutic treatments with IL-17 blockers (i.e., neutralizing anti-IL-17 antibody, IL-17 receptor IgG1 Fc fusion protein, and vaccination against IL-17) resulted in reduced chronic inflammation and cartilage degradation in both CIA and AIA models (44–47). Based on these data from the 3 animal models, can we draw a conclusion that IL-17 plays a pivotal role in both initiating phase and ongoing phase of RA? Of course, it is not true, since numerous clinical studies confirmed that RA patients failed to exhibit a satisfactory response to neutralization of IL-17/IL-17 receptor (i.e., secukinumab, ixekizumab, and brodalumab) (48–51).

Taken together, the available data from one animal model are not able to support that IL-23 is involved in an initiating rather than ongoing phase of axSpA.

## Th17 Cells Are Not Principal Inflammatory Cells in the Pathogenesis of Axial Spondyloarthritis

In 2005, two groups independently discovered a new specific subset of CD4+ T cells, Th17 cells, which mainly produce IL-17 (52, 53). Except for IL-17, Th17 cells also produce IL-22 and certain other proinflammatory cytokines.

IL-23 significantly enhanced IL-17 secretion (54), and Th17 cells were absent in IL-23-knockout mice (55). These data imply that IL-23 is critical for Th17 cell development or survival. Later studies discovered the combination of IL-6 and transforming growth factor (TGF)-β induced transcription factor RORγt in naïve T cells and upregulated IL-23R expression (56). IL-23/IL-23R signaling plays an important role in the stabilization of the Th17 phenotype and expansion of Th17 cells (56). In human CD4+ T cells, IL-1β induced differentiation of Th17 cells, and IL-6 further enhanced the differentiation (57). So the differentiation, expansion, and stabilization of human Th17 cells depend on the presence of TGF-β, IL-23, IL-6, and IL-1β (58). Thereafter, the IL-23/IL-17 axis has been attracting a lot of attention in inflammatory disorders, including axSpA (59).

The finding from genome-wide association studies (GWAS) was a major stimulus to consider the importance of Th17 cells in axSpA or AS, which demonstrated that polymorphisms in multiple genes of those cytokines and their signaling pathways are involved in the Th17 pathway, such as *IL-23R*, *IL-12B* (encoding p40 of IL-23 and IL-12), *IL-1R2*, *IL-6R*, and *RUNX3 (*60, 61). In 2008–2009, increased proportions of Th17 cells in peripheral blood were found in AS patients (62, 63). Moreover, the protective effect to develop AS was found in the carriers with *R381Q* IL23 receptor polymorphism, resulting in a lower percentage of circulating Th17 cells (64, 65). All these data indirectly support that Th17 cells play an important role in the pathophysiology of AS. However, the evidence from clinical trials to block the cytokines in the differentiation or expansion of Th 17 cells is disappointing. As discussed above, IL-23 inhibition with both ustekinumab targeting IL-23p40 and IL-12p40, and risankizumab targeting IL-23p19, failed to exert significant efficacy in AS (14, 15). Neither tocilizumab, a humanized monoclonal antibody against IL-6 receptor, nor sarilumab, a fully human monoclonal antibody anti-IL-6R, showed satisfactory efficacy in patients with active AS or SpA (66, 67). IL-1 inhibition with anakinra, a recombinant IL-1 receptor antagonist, failed to show satisfactory improvement in AS either (68).

These data imply that Th17 cells are not principal inflammatory cells in the pathogenesis of axSpA, which does not mean that IL-17 is not a principal proinflammatory cytokine in axSpA, because Th17 cells are not the sole cell source of IL-17 (3).

## IL-17 Can Be Produced by Several Immune Cell Types Other Than Th17 Cells in Axial Spondyloarthritis Independently of IL-23

The IL-17 superfamily consists of six members (IL-17A to IL-17F). IL-17A and IL-17F are the two members with the highest sequence homology to each other (50%) and share similar actions. IL-17 acts on many cell types including T cells themselves. In inflammatory arthritis, IL-17A can induce the production of IL-1β, IL-6, TNF-α, C-C motif chemokine ligand 2 (CCL2), matrix metalloproteinases (MMPs) (including MMP1, MMP9, and MMP13), and receptor activator of nuclear factor-κB (NF-κB) ligand (RANKL) from target cells, thereby perpetuating the inflammation, driving the degradation of extracellular matrix, and inducing bone erosion within the joint. IL-17 also promotes angiogenesis, thus increasing blood flow and facilitating the influx of inflammatory cells into the inflamed joint [reviewed in (69)].

Increased expression of IL-17A was found in the synovial fluid and/or serum from patients with active AS (70). In AS facet joints, the significantly increased frequency of IL-17-secreting cells was demonstrated (71). These data suggest that IL-17 might participate in the pathophysiology of AS.

However, immunohistological analysis further revealed that the majority of IL-17(+) cells in AS facet joints were neutrophils, while CD3+ T cells and AA-1+ mast cells were less often IL-17-positive (71). No significant difference in the frequency of Th17 cells in the blood was found between axSpA patients and healthy controls (71). These data suggest that Th17 cells might be not the major source of IL-17 in axSpA/AS, although IL-17 was first thought to be secreted by CD4+ Th17 cells.

Recently, IL-17 has been demonstrated to be produced by lymphocytes of both the adaptive and innate immune systems. Aside from Th17 cells, IL-17-producing CD8+ T cells (Tc17) also produce IL-17 (72). During an inflammatory response, much of the IL-17 is produced by innate immune cells, including γδ T cells (73), mucosal-associated invariant T (MAIT) cells (74), natural killer (NK) cells (75, 76), invariant NK T (iNKT) cells (77), type 3 innate lymphoid cells (ILC3) (78), lymphoid tissue inducer (LTi) cells (79, 80), and neutrophils (81). RORγt, the master transcriptional regulator of Th17 cells, is also expressed by IL-17-producing innate-like T cells, such as iNKT and γδ T cells (82).

Increased levels of conventional CD8+ Tc17 cells were found in the blood of AS patients, and the proportion of Tc17 cells positively correlated with the disease severity of AS. AS patients had a lower frequency of γδ T cells and MAIT cells in the peripheral blood but had an elevated frequency of IL-17A+ MAIT cells in blood as compared with healthy controls (83–85). Compared with RA, there are more enriched IL-17A+ MAIT cells in the synovial fluid of AS (83, 84). Expression of a MAIT cell activation marker CD69 on IL-17A+ MAIT cells was correlated with the Ankylosing Spondylitis Disease Activity Score (ASDAS) in patients with AS (84). In normal human spinous processes, entheseal soft tissue, and peri-entheseal bone, entheseal γδ T cells with the phenotype of Vδ1 and Vδ2 subsets were confirmed immunohistochemically (86). IL-17-producing ILC3 cells were expanded in the blood, synovial fluid, gut, and bone marrow of patients with AS (87). Increased IL-17+ NK, γδ T, and ILC3 cells in peripheral blood and synovial fluid were found in patients with reactive arthritis, enteropathic SpA, and undifferentiated SpA (88, 89). All these data support that the IL-17-producing cells may be involved in the pathophysiology of SpA. Furthermore, upregulation of IL-17 in AS MAIT cells was not dependent on priming with IL-23 (83). In γδ T cells, the production of IL-17A was also IL-23-independent (86, 90).

Taken together, IL-17 may be produced independently of IL-23 in the immune cells other than Th17 cells in axSpA.

## IL-23 Overexpression Is Not Sufficient to Induce Enthesitis and Bone Formation

IL-23 plays a key role in amplifying and maintaining IL-17 production in many cells. Monocytes and dendritic cells are the primary cells releasing IL-23. A CD14+ myeloid population (monocytoid cells) in the human enthesis was found to produce IL-23, IL-1β, TNF, and CCL20 (91). In the facet joints of patients with AS, IL-23-positive cells were found to be increased in the subchondral bone marrow, but not increased in fibrous tissue (92). In 2007, *IL-23R* gene polymorphisms were first revealed as risk factors for developing AS (93). In 2012, *IL-12B* (encoding p40 of IL-23 and IL-12) gene polymorphism was found to be associated with the development of AS (94). Elevated serum IL-23 levels in AS were found in 2 pilot studies (95, 96). These data indirectly suggest the pathogenic role of IL-23 in AS. But there were also conflicting data regarding the higher susceptibility of *IL-23R* gene polymorphisms (97, 98) and a higher serum IL-23 level in active AS (99).

In 2011, Adamopoulos et al. (100) reported that the phenotype of the animal model with systemic overexpression of IL-23 was characterized with chronic arthritis and severe bone loss. The animal model with systemic overexpression of IL-23 was induced by hydrodynamic delivery of an IL-23 minicircle DNA into the tail veins of B10.RIII mice, and serum IL-23 was stably expressed for at least 90 days. Histological analysis of the inflamed paws and knees revealed synovitis, pronounced neovascularization/panus, myelopoiesis in the bone marrow, and extensive erosion with numerous osteoclasts. IL-23-mediated structural damage to the skeleton and extensive erosion of cortical bone were also noted by micro-CT. Compared with wild-type mice, although there were equal numbers of osteoclast precursors in bone marrow monocytes isolated from IL-23p19^−/−^ mice, the IL-23p19^−/−^ osteoclast precursors had impaired ability to differentiate into mature osteoclasts and had impaired ability of dentine resorption (100). *In vitro*, IL-23 was demonstrated to promote osteoclastogenesis in a lot of studies (56, 101–105), and IL-23 might indirectly inhibit osteoclast differentiation *via* activated T cells (106, 107). These findings cannot account for the pathophysiological role of IL-23 in axial SpA and did not catch enough attention. On the contrary, in 2012, the findings from another study with the same animal model satisfied our expectation (108) and rapidly attracted numerous scientists’ attention (109, 110).

In this attractive study (108), systemic overexpression of IL-23 in B10.RIII mice was also induced by hydrodynamic delivery of an IL-23 minicircle DNA into the tail veins, which resulted in long-term expression of IL-23 and elevated serum IL-23 for at least 100 days. The mice with IL-23 overexpression developed severe paw swelling. Histological analysis revealed severe entheseal inflammation, and such enthesitis was presently accompanied with new entheseal bone but no synovial joint destruction (108). The evident new bone formation in the paws of the IL-23-overexpression mice was further confirmed by high-resolution micro-CT. In the entheseal cells purified from the animal model, IL-23 promoted IL-17 and IL-22 expression. IL-22 promoted entheseal and periosteal bone formation by phosphorylation of STAT3 in osteoblasts (108). IL-23 was demonstrated to be essential and to act on RAR-related orphan receptor γt (ROR-γt)+CD3+CD4−CD8− entheseal resident T cells to induce enthesitis (108). These findings perfectly interpreted the pathophysiology of SpA to a great extent. However, the animal model with enthesitis induced by IL-23 overexpression has never been replicated by other research groups. On the contrary, later studies confirmed that the animal model with systemic overexpression of IL-23 presented peripheral arthritis with histological synovitis but no spinal inflammation or enthesitis (111). In addition, T cells are not essential for the development of enthesitis in TNF^ΔARE^ mice (deleting TNF AU-rich elements (ARE) from the mouse genome on the regulation of TNF biosynthesis) (112) and entheseal ossification in an aged DBA/1 mouse (113). Furthermore, our results demonstrated that the mRNA expression of IL-22 receptors and IL-23R was not detectable in 3 osteoblastic cell lines (i.e., C2C12, MC3T3-E1, and Saos-2 cells) and primary osteoblasts isolated from bone marrow, even after bone morphogenetic protein-2 stimulation (114). Neither IL-22 nor IL-23 showed any significant effects on primary osteoblasts, including the cell proliferation, alkaline phosphatase activity, and mRNA expression of alkaline phosphatase, osteocalcin, and Runt-related transcription factor 2 (Runx-2). The null effect of IL-23 on osteoblasts was confirmed by other research groups (107). Up to now, there are no other studies confirming the stimulatory effects of IL-22 on osteoblasts, which was reported in 2012 (108).

Taken together, there were no solid and replicable data to demonstrate that IL-23 overexpression could induce enthesitis and bone formation and that IL-22 could stimulate osteoblasts.

## In Summary

The IL-23/IL-17 axis was widely accepted as an important pathway in the pathogenesis of axSpA. IL-23 was an upstream cytokine to facilitate Th17 cell expansion and then to increase IL-17 production. When good efficacy of IL-17 inhibitors has been demonstrated in axSpA, it was reasonable to expect that IL-23 inhibition would exert similar efficacy with IL-17 inhibition. Unfortunately, IL-23 inhibitors failed in the treatment of axSpA, although both IL-17 inhibitors and IL-23 inhibitors succeeded in the clinical trials of psoriasis. All the blockers of IL-6 and IL-1β, which also mediate the differentiation of Th17 cells, failed to exert satisfactory effects in axSpA either. Recently, mounting evidence shows that γδ T cells, MAIT cells, NK cells, iNKT cells, and neutrophils might produce IL-17 independently of IL-23. Furthermore, the evidence supporting IL-23 overexpression to induce enthesitis and bone formation cannot be replicated and is conflicted with the results of other research groups. Up to now, there is no solid evidence to support IL-23 is involved in the pathophysiology of axSpA.

## Author Contributions

HZ and H-LJ drafted the manuscript, and S-MD conceived the study and edited the manuscript. All authors listed have made a substantial, direct, and intellectual contribution to the work and approved it for publication.

## Funding

This project was supported by a grant from the National Natural Science Foundation of China (No. 82071809).

## Conflict of Interest

The authors declare that the research was conducted in the absence of any commercial or financial relationships that could be construed as a potential conflict of interest.

## Publisher’s Note

All claims expressed in this article are solely those of the authors and do not necessarily represent those of their affiliated organizations, or those of the publisher, the editors and the reviewers. Any product that may be evaluated in this article, or claim that may be made by its manufacturer, is not guaranteed or endorsed by the publisher.
